# On-chip optical vortex-based nanophotonic detectors

**DOI:** 10.1038/s41377-020-00359-8

**Published:** 2020-07-03

**Authors:** Alina Karabchevsky

**Affiliations:** School of Electrical and Computer Engineering, Ben-Gurion University of the Negev, Beer-Sheva, 8410501 Israel

**Keywords:** Sub-wavelength optics, Integrated optics

## Abstract

An on-chip optical vortex detector based on spin-Hall nanoslits is reported. The detector is sensitive to the spin of the incoming beam and can simultaneously record the polarization and phase singularity. Although the reported device relies on fast decaying surface plasmons, it represents an important step forward in the development of optical vortex-based integrated photonic devices.

The ever-growing demand for compact integration has given rise to integrated photonic devices on different material platforms for emerging applications, including on-chip biosensors, on-chip quantum technologies and, recently, even on-chip optical vortex-based detectors with plasmonic materials^1–3^.

An *optical vortex* is a beam of light that propagates such that the phase experiences phenomenological *singularity* and the wavefront has a topological structure with topological charge due to the helicoidal spatial wavefront around this phase singularity. Such a topological structure can be found in many disciplines, including optics, acoustics, and others. To understand the influence of the singularity, one must explore the phase, polarization, and amplitude of the incident beam. While exploring these properties of the special beam, one may discover that both the polarization and the amplitude vanish and the phase cannot be determined. These insights were published as a new concept in wave theory in 1974. In their theoretical work, Nye and Berry reported on the observation of so-called dislocations^4^.

The first on-chip silicon-integrated optical vortex *emitter* was reported in the journal *Science* in 2012^5^. Having an emitter and a detector monolithically integrated on the same chip would be an essential step forward for on-chip optical vortex-based photonic devices. In their experimental work, Feng and coworkers developed a novel on-chip optical *detector* that allows full characterization of the polarization and phase singularity based on a plasmonic spin-Hall nanograting. Their method of detection is different from that in other state-of-the-art systems^6^, because it allows for the detection of singularities simultaneously. Interestingly, the detector developed by Feng and coworkers does not require complicated alignment. It is based on an asymmetric metallic array, in which the top and bottom parts of the array have different grating constants. The specific surface topology dictates the angle at which the excited surface plasmon polariton (SPP) will propagate. The asymmetry allows differentiation of the sign of the topological charge (phase), while the spin-Hall slits are sensitive to the spin of the incoming beam. The so-called spin-Hall slits are composed of nanoslits oriented at π/2. This orientation of the nanoslits gives rise to a chiral response of the detector. If the slits are reversed, then the inverted chiral response occurs. This special design allows the authors to distinguish between a left-circularly polarized (LCP) beam and a right-circularly polarized (RCP) beam. Simply put, a beam incident on the detector would excite an SPP that travels at an angle *θ* to one of the four quadrants, as shown in Fig. 1. The quadrant in which the SPP propagates determines the polarization (RCP/LCP) of the beam and the sign of the topological charge *l*. The angle *θ* dictates the magnitude of the topological charge.

**Fig. 1:**
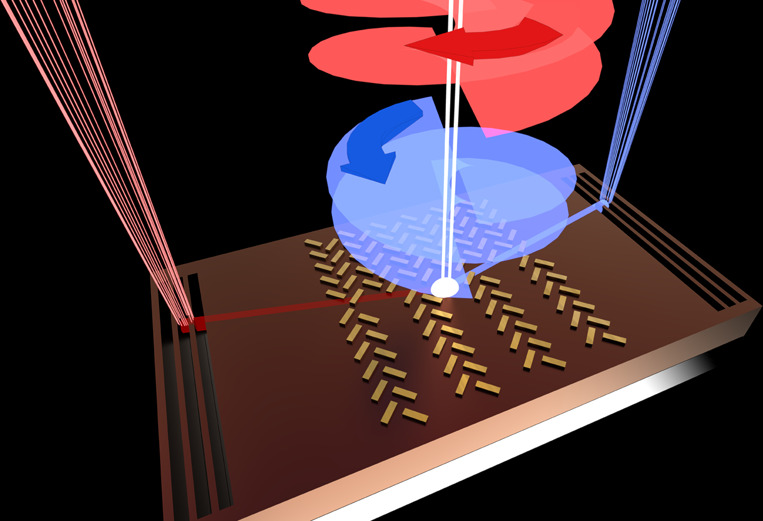
Artistic impression of the on-chip optical vortex detector that records the phase and polarization singularities. Two orbital angular momentum beams with opposite polarization states and different topological charges illuminate the on-chip metasurface.

The great advantage of such devices is their ultracompact size and extremely simple operation^2,3^, which make them easy to integrate with other on-chip components, e.g., modulators and lasers, to form photonic integrated circuits, enabling large-scale integrated photonic applications.

The next milestone to achieve in optical vortex-based integrated photonic devices is to demonstrate the operation by eliminating the inevitable ohmic losses of plasmonic materials and exploring other surface waves such as Bloch waves, which in turn can propagate on lossless dielectric materials. One can expect significant advances in integrated photonics in the coming years in the direction of novel optical vortex-based on-chip devices.
